# Microbial Electricity Generation Enhances Decabromodiphenyl Ether (BDE-209) Degradation

**DOI:** 10.1371/journal.pone.0070686

**Published:** 2013-08-05

**Authors:** Yonggang Yang, Meiying Xu, Zhili He, Jun Guo, Guoping Sun, Jizhong Zhou

**Affiliations:** 1 Guangdong Provincial Key Laboratory of Microbial Culture Collection and Application, Guangdong Institute of Microbiology, Guangzhou, China; 2 Institute for Environmental Genomics and Department of Botany and Microbiology, University of Oklahoma, Norman, Oklahoma, United States of America; 3 State Key Laboratory of Applied Microbiology, South China (The Ministry-Province Joint Development ), Guangzhou, China; 4 Earth Sciences Division, Lawrence Berkeley National Laboratory, Berkeley, California, United States of America; Missouri University of Science and Technology, United States of America

## Abstract

Due to environmental persistence and biotoxicity of polybrominated diphenyl ethers (PBDEs), it is urgent to develop potential technologies to remediate PBDEs. Introducing electrodes for microbial electricity generation to stimulate the anaerobic degradation of organic pollutants is highly promising for bioremediation. However, it is still not clear whether the degradation of PBDEs could be promoted by this strategy. In this study, we hypothesized that the degradation of PBDEs (e.g., BDE-209) would be enhanced under microbial electricity generation condition. The functional compositions and structures of microbial communities in closed-circuit microbial fuel cell (c-MFC) and open-circuit microbial fuel cell (o-MFC) systems for BDE-209 degradation were detected by a comprehensive functional gene array, GeoChip 4.0, and linked with PBDE degradations. The results indicated that distinctly different microbial community structures were formed between c-MFCs and o-MFCs, and that lower concentrations of BDE-209 and the resulting lower brominated PBDE products were detected in c-MFCs after 70-day performance. The diversity and abundance of a variety of functional genes in c-MFCs were significantly higher than those in o-MFCs. Most genes involved in chlorinated solvent reductive dechlorination, hydroxylation, methoxylation and aromatic hydrocarbon degradation were highly enriched in c-MFCs and significantly positively correlated with the removal of PBDEs. Various other microbial functional genes for carbon, nitrogen, phosphorus and sulfur cycling, as well as energy transformation process, were also significantly increased in c-MFCs. Together, these results suggest that PBDE degradation could be enhanced by introducing the electrodes for microbial electricity generation and by specifically stimulating microbial functional genes.

## Introduction

Polybrominated diphenyl ethers (PBDEs) are a class of flame retardants that have caused considerable concern in recent years due to their elevated levels of detection in the environment, particularly human tissues [1], [2]. Penta-BDE, octa-BDE and deca-BDE are three major commercial PBDE mixtures. The former two products have been included on the Stockholm convention list of priority persistent organic pollutants and banned from the European market since 2003 and effectively eliminated in North America since 2005 [3]. However, deca-BDE, which is mainly composed of deca-BDE (BDE-209) and a small amount of nona-BDEs, accounts for approximately 83% of the worldwide use of PBDEs, and is still legally used in most countries. As a result, BDE-209 was detected as the substantially dominant PBDE congeners in sediment environments [4]. Recent studies demonstrated that BDE-209 could be anaerobically debrominated by microorganisms [5], [6], [7]. However, the degradation of these highly persistent halogenated compounds is very slow and always produces toxic lower brominated PBDE congeners under anaerobic conditions [8].

It has been reported that the amendments of co-substrates [7], primer compounds [5] or electron donors [9] could stimulate PBDE biodebromination. However, the main problem for PBDE biodegradation is the low biomass and activity of the functional microbes. Researchers have found that the lack of suitable electron acceptors was the main limitation for organic pollutant degradation under anaerobic condition and the supplementary of electron acceptors could even avoid the production of toxic daughter products by providing necessary electron flow to stimulate the viabilities of indigenous microorganisms [10]. Under aerobic conditions, oxygen is the preferential electron acceptor. However, under anaerobic conditions, electron acceptors are usually limited and the microbial metabolic activities are depressed. In order to stimulate indigenous microbial growth and activity/viabilities, alternative terminal electron acceptors to oxygen, such as nitrate, sulfate and ferric iron were employed in anaerobic bioremediation processes [10], [11]. With the development of microbial fuel cells (MFCs), electrodes were introduced as the solid terminal electron acceptor to stimulate anaerobic biodegradation [12], [13]. In the anode compartment of bioelectrochemical system (BES), microorganisms can respire with anode by converting the energy stored in chemical bonds in organic compounds to generate electricity. Such an electron transfer is highly promising for bioremediation of organic pollutants, such as petroleum hydrocarbons, wastewaters, and contaminated soils and sediments. More and more studies have demonstrated that electrodes can served as electron acceptors for stimulating the anaerobic degradation of organic pollutants in contaminated environments, including petroleum hydrocarbons [13], [14] and chlorinated compounds [15].

Since the structures between polychlorinated biphenyls (PCBs) and PBDEs are very similar, it is likely that some dechlorinating enzymes may be involved in PBDE transformation [16], [17]. However, little is known about the microbial transformation of PBDEs, especially enzymes necessary for PBDE biodegradation. Our previous studies found that the structure and composition of PBDE-degrading microbial communities could be affected by the environmental parameters, and that most commonly genera involved in PBDE degradation were anodic respiring bacteria, such as *Geobacter*, *Shewenalla*, and *Pseudomonas* [9], [18]. It is necessary to identify key functional genes and their associated populations involved in PBDE degradation so that an effective bioremediation strategy can be developed.

In this study, we hypothesized that the introduction of extra electron acceptor through the external circuit would stimulate the diversity and abundance of microbial communities and accelerate PBDE biodegradation. To test those hypotheses, two different operating modes were conducted in dual-chamber MFC systems, one operated under closed-circuit condition (c-MFC) and the other under open-circuit condition (o-MFC) as controls were designed for PBDE degradation. A functional gene array, GeoChip 4.0, which contains 120,054 distinct probes, covering 200,393 coding sequences (CDS) for genes in different processes, was used to examine the impacts of concomitant electricity production on the functional microbial community composition and structure, and the linkages between microbial community structures and PBDE degradation rates. Our results indicated that microbial electricity generation dramatically increased microbial community functional gene diversity and abundance, especially those genes involved in chlorinated solvent remediation and aromatic hydrocarbon degradation, which were correlated with BDE-209 degradation.

## Materials and Methods

### Reactor Setup and Operational Conditions

Two differently operating modes (closed-circuit and open-circuit) were conducted in dual-chamber MFC systems which were assembled as previously described [19]. The anodic compartments contained 200 ml of modified M9 medium supplemented with 20 mM lactate (electron donor) and 1.4 µM BDE-209. Prior to the addition of defined medium, BDE-209 resolved in dichloromethane was added to each anodic compartment and evaporated in the dark. The cathode compartments were filled with 200 ml of *phosphate buffered saline* (PBS) solution containing 50 mM ferricyanide. The BDE-209-degrading enrichment [9] was inoculated to anodic chambers to start the c-MFC and o-MFC operation. All of the MFC systems were incubated at 30°C in the dark without agitation, after the anode compartments were purged with pure nitrogen gas for 5–10 min. Voltage output of the c-MFC under an 800 ohm external resistor was recorded every 4 min using a multimeter (UT71D, Uni-Trend Technology). Lactate was re-supplemented to the systems when the voltage in c-MFC decreased to its lowest value. Anodic cultures in these two kinds of MFC systems were periodically sampled and used for subsequent analyses. The concentrations of unbounded bromine ion in anodic culture were determined using an ion chromatography system (ICS1500, Ionpac AS19/AG19, Dionex) and a gradient KOH eluent (10 mM for 0–10 min, 10–45 mM for 10–25 min, 45 mM for 25–30 min, 10 mM for 30–37 min). After 70-day performance, BDE209-degrading rates and the PBDE congener profiles were analyzed as previously described [9]. In brief, the whole anodic chambers including the culture liquids and anodes were extracted with dichloromethane for 3 times. The resulting extracts were evaporated in a rotary evaporator and re-dissolved with *n-*hexane. PBDE congeners dissolved in *n-*hexane were then analyzed by a Shimadzu Model 2010 gas chromatograph (GC) coupled with a Model QP200 mass spectrometer (MS). Standard curves for the quantitative determination of PBDEs were prepared using a gas chromatograph-electron capture detection (GC-ECD).

Sample collections and the subsequent treatments were conducted in triplicate.

### DNA Preparation and GeoChip 4.0 Analysis

DNA was extracted using the TIANamp Bacteria DNA Kit (TIANGEN BIOTECH (BEIJING) CO., LTD.) according to the manufacturer’s instructions. DNA amplification and labeling, as well as the purification of labeled DNA, were carried out according to the methods described by Xu *et al.* [20]. The GeoChip 4.0 synthesized by NimbleGen (Madison, WI, USA) was used to analyze the functional structure of the microbial communities. All hybridizations were carried out at 42°C with 40% formamide for 16 h on a MAUI hybridization station (BioMicro, Salt Lake City, UT, USA). After hybridization, the arrays were scanned with a NimbleGen MS200 microarray scanner (Roche NimbleGen, Madison, 113 WI, USA) at a laser power of 100% and 100% PMT (photomultiplier tube). Signal intensities were measured based on scanned images, and spots with signal-to-noise ratios lower than 2 were removed before statistical analysis as described previously [21].

### Statistical Analysis

Pre-processed GeoChip data were further analyzed with different statistical methods: (i) cluster analysis was performed using the pairwise average-linkage hierarchical clustering algorithm in the CLUSTER software (http://rana.lbl.gov/EisenSoftware.htm) for microbial community structure and composition, and the results of hierarchical clustering were visualized using TREEVIEW software (http://rana.lbl.gov/EisenSoftware.htm); (ii) principal component analysis (PCA) performed by CANOCO 4.5 for Windows (Biometris – Plant Research International, The Netherlands), and three different non-parametric analyses performed with the Vegan package (v.1.17–12) in R v. 2.13.1 (R Development Core Team, 2011): analysis of similarities (ANOSIM), non-parametric multivariate analysis of variance (Adonis) and Multi-Response Permutation Procedure (MRPP), were conducted to determine the overall functional gene changes in the microbial communities; (iii) microbial diversity index analyzed by Krebs/win version 0.94 (http://www.biology.ualberta.ca/jbrzusto/krebswin.html), Significant Pearson’s linear correlation (*r*) analysis and analyses of variance (ANOVA) analyzed by SPSS 16.0 for Windows and response ratio (RR) (Data S1 in File S1) using the formula described by Luo et al. [22] for identifying the genes with significant change; (iv) canonical correspondence analysis (CCA) for revealing the individual or set of environmental variables that significantly explained the variation in functional microbial communities [23], [24]; and (v) partial CCA were calculated using functional gene communities and PBDE congener compositions and hydrochemical parameters for each treatment as covariables. All the analyses were performed by functions in CANOCO 4.5 for Windows (Biometris – Plant Research International, The Netherlands).

## Results

### Bioreactor Performance

**Figure 1:**
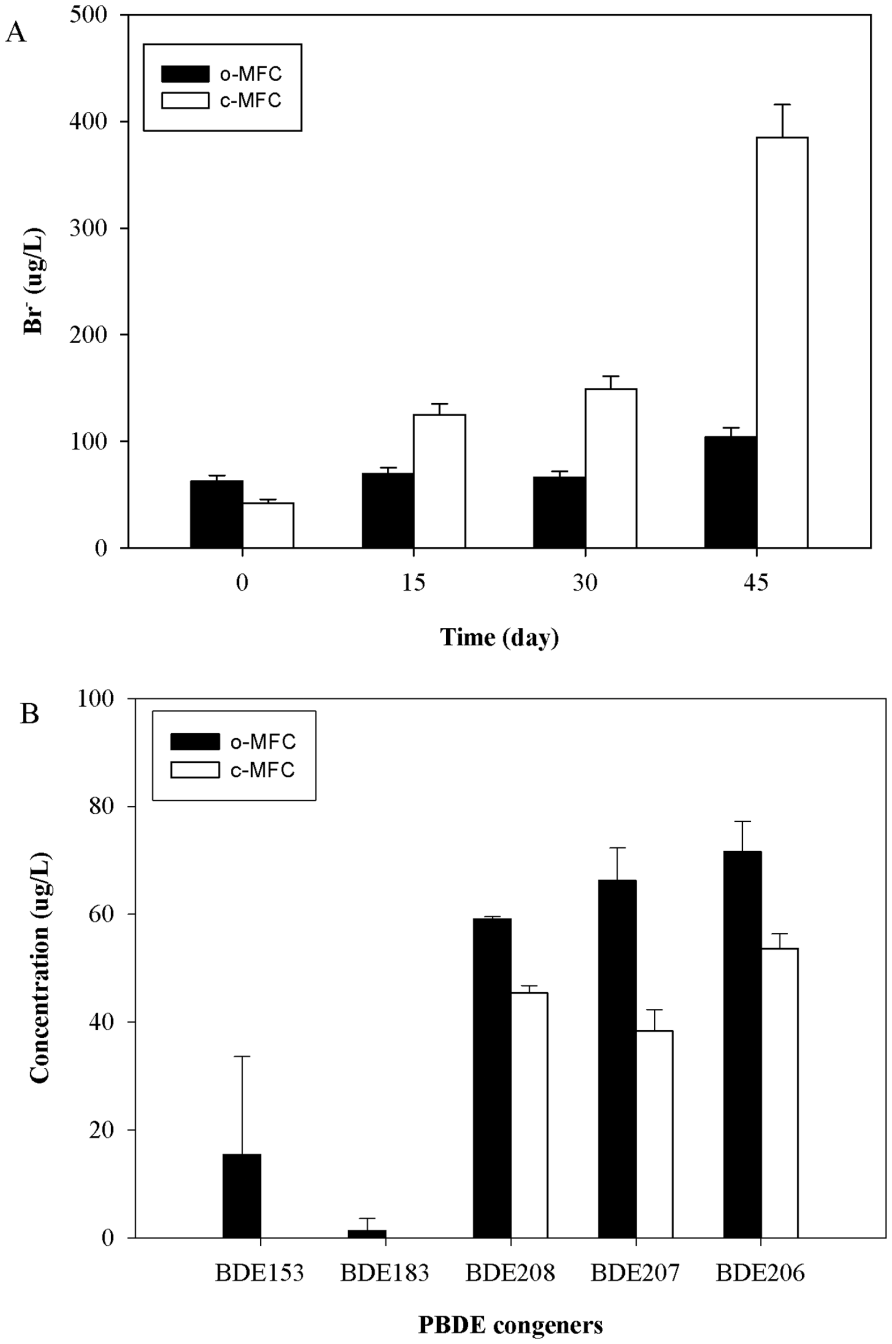
PBDE congener products of BDE-209 after 70 days performance by c-MFCs and o-MFCs.

The voltage of c-MFC under the external resistor increased to 0.41 V after 12 days performance, and was subsequently decreased with the consumption of electron donor. Supplement of lactate could immediately recover current generation. The maximum voltage increased from 0.41 V to 0.46 V in the first two current generation periods and then showed a slight decrease in the following periods (Figure S1A in File S1). Polarization analysis showed a maximum power density of 3.3 W/m^3^ (0.28 W/m^2^) and an internal resistance of 325 ohm for the c-MFCs (Figure S1B in File S1). After 45-day performance, the concentrations of unbounded bromine ions in c-MFCs and o-MFCs were 385.0±30.8 µg/L and 104.4±8.4 µg/L (Figure 1A), respectively. On day 70, significantly (*p*<0.01) different BDE-209 degradation rate were detected with around 58.9% in c-MFCs and 23.2% in o-MFCs. Obviously different PBDE congener profiles were observed between c-MFCs and o-MFCs. The concentrations of three nona-BDE congeners, BDE-208, -207, -206, were commonly found in both systems with a significant decrease in c-MFCs (Figure 1B), while the lower brominated PBDE congeners, BDE-153 and BDE-183 were only detected in o-MFCs. The concentrations of BDE-209 and the less brominated PBDE products were much lower in c-MFCs, suggesting that the PBDE removal could be markedly improved under microbial current generation condition.

### Effects of Microbial Electricity Generation on the Overall Functional Structure of Anode Microbial Communities

To assess the effects of microbial electricity generation on the overall functional structure of anode microbial communities, the anode microbial community functional compositions and structures on day 70 were analyzed using GeoChips 4.0. Among a total of 9111 genes detected, 5646.5±9.2 were from c-MFC systems and only 638.3±56.9 were from o-MFC systems with 87.9% unique to c-MFC samples. For the shared genes, around 12.6% was significantly (*p*<0.01) increased and only 0.7% was significantly (*p*<0.01) decreased in c-MFC systems. The overall microbial functional diversity was significantly (*p*<0.02) higher in c-MFC based on the Shannon-Weiner (*H*’) index. The functional gene numbers and abundances of all functional gene categories were also significantly (*p*<0.01) increased in c-MFCs by computing the response ratio (RR). The total number and abundance of genes detected in c-MFCs were 9.7 and 8.5 times higher than those in o-MFCs, respectively (Table S1 in File S1).

**Figure 2:**
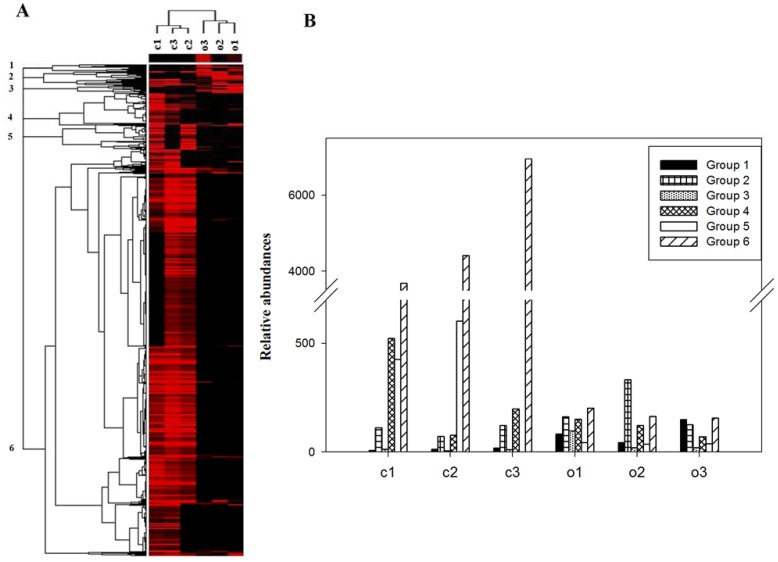
Cluster analysis of functional genes detected using GeoChip 4.0. The figure was generated using CLUSTER and visualized in TREEVIEW. Black indicates signal intensities below the threshold value and red indicates a positive hybridization signal. The color intensity indicates differences in signal intensity. The samples from c-MFCs and o-MFCs on day 70 were clearly separated in two groups. Six different gene patterns were observed and indicated by numbers in the tree (A), and also illustrated in the graphs (B). (c1, c2, c3 represented the three replications of c-MFCs and o-1, o-2, o-3 represented those from o-MFCs.).

Hierarchical clustering analysis showed that the c-MFC samples were clustered together and well separated from the o-MFC samples and six major gene groups could be visualized (Figure 2A). Within these six groups, 82.7% of detected genes belonged to Group 6, following with Group 4 (7.3%), Group 5 (5.0%), Group 2 (2.8%), Group 1 (1.5%) and Group 3 (0.7%) (Figure 2B). In Group 6, more than 92.3% genes detected were contributed by the c-MFC samples and the total gene abundance from c-MFCs was 28.9 times higher than that from o-MFCs with significant (*p*<0.01) increases in different gene categories as the following trend: energy process (15.7)<metal resistance (26.3) <phosphorus (27.1)<nitrogen cycling (27.3)<stress (28.6) = organic remediation (28.6)<carbon cycling (33.6)<sulphur (59.8). Within the 550 species detected in Group 6, 381 species were unique to the c-MFC samples.

The abundances of most genes involved in carbon (C), nitrogen (N), phosphorus (P) and sulfur (S) cycling showed significant increases in c-MFCs. Among the C cycling genes detected, more than 86.2% involved in carbon degradation and almost all of these genes showed significantly (*p*<0.05) higher abundances in c-MFCs than in o-MFCs (Figure S2 in File S1). Totally, 745 N cycling genes were detected and belonged to 15 gene families involved in assimilatory N reduction, dissimilatory N reduction to ammonium, N_2_ fixation, denitrification processes, and ammonification (Figure S3 in File S1). Among those 15 gene families detected, 12 of them had significantly (*p*<0.05) higher abundances in c-MFCs than in o-MFCs. Higher number and abundance of the genes involved in S cycling were detected in c-MFCs (Figure S4 in File S1). For the detected 113 *dsrA/B* genes encoding dissimilatory sulfite reductase, 98 were unique to c-MFCs and four were unique to o-MFCs. For the genes involved in P cycling, 86 genes were detected and more than 82.1% of them were unique to c-MFC systems. The total abundance of *ppk* genes encoding polyphosphate kinase in c-MFCs was significant (*p*<0.05) higher than that in o-MFCs (Figure S5 in File S1). Details for these genes and their changes in c-MFC and o-MFC systems are described in the Data S2 in File S1.

All detected genes were derived from 574 genera with around 51.2% unique to c-MFCs and only 1.4% unique to o-MFCs. For the total signal intensities of the commonly detected genera, 11 were significantly increased at the *p*<0.01 level, and 72 were significantly increased at the *p*<0.05 level in c-MFCs. The total signal intensity of most well known versatile anodic respiring genera, *Geobacter* spp., *Shewanella* spp., *Pseudomonas* spp., and *Desulfovibrio* spp., were significantly enriched in c-MFCs at the *p*<0.01 or *p*<0.05 level, while no significant difference in the total signal intensity was observed for known dehalogenating microorganisms, such as *Dehalococcoides* spp. [25] and *Desulfuromonas* spp. [26].

### Stimulations of the Functional Genes Involved in Aromatic Hydrocarbon Degradation and Chlorinated Solvent Remediation

**Figure 3:**
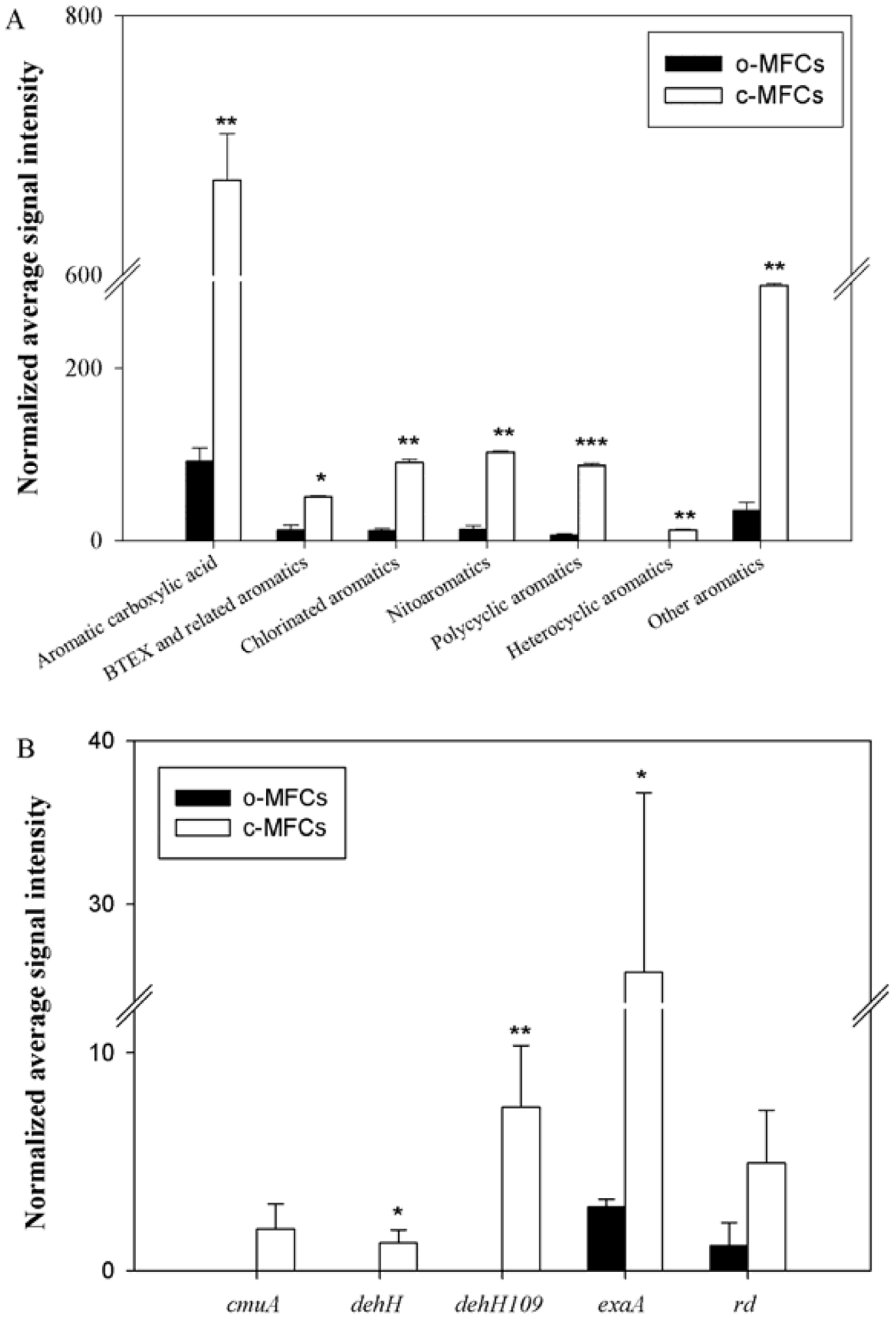
The normalized signal intensity of the detected key gens families involved in aromatic compound degradation (A) and chlorinated solvent remediation (B) under both c-MFCs and o-MFCs. The signal intensities were the sum of detected individual gene sequences for each functional gene, averaged among three samples. All data are presented as mean±SE. *** *p*<0.001, **p<0.05, *p<0.10.

Some previous studies have shown that dioxygenases for catalyzing the oxidation of aromatic compounds were responsible for PBDE transformation [16], [17]. Consistently, GeoChip results showed that more than 75.5% of the organic remediation genes detected were involved in aromatic degradation and the aromatic degradation gene numbers detected in c-MFCs was around 10.5 times of those in o-MFCs. The abundances of relative genes for polycyclic aromatics, aromatic carboxylic acid, chlorinated aromatics, nitroaromatics and other aromatics showed significant (*p*<0.05) increases in c-MFCs (Figure 3A), and the genes for heterocyclic aromatics degradation were only detected in c-MFCs. Among the 45 aromatic degradation genes with significant changes in RR (Table S2 in File S1), only two of them, *pimF* from *Bordetella petrii* (163261940) for aromatic carboxylic acid degradation and *nahA* from *Caulobacter* sp. K31 (167645881) for polycyclic aromatics degradation, showed decreases under electricity generation conditions. The significant stimulation of the aromatic degradation genes suggests that these genes may be involved in PBDE transformation, and that the aromatic hydrocarbons transferred from BDE-209 could be further degraded under current generation condition.

Furthermore, GeoChip detected 52 chlorinated solvent remediation genes from a variety of microorganisms with 48 unique to c-MFCs, 2 unique to o-MFCs and one shared by both. The reductive dehologenase gene (*rd*) from *Dehalococcoides ethenogenes* 195 (57233751) was shared by both. The two unique genes to o-MFCs were the pyrroloquinoline quinone (PQQ)-dependent ethanol dehydrogenase gene (*exaA*) from *Gluconacetobacter diazotrophicus* PAl 5 (162146364) and the reductive dehologenase gene (*rd*) from *Dehalococcoides* sp. VS (163811600). The 48 unique chlorinated solvent remediation genes detected in c-MFCs belonged to five gene families, including *cmuA* for methyltransferase, *dehH* for hydrolase, *dehH109* for haloacid dehalogenase, *exaA* for pyrroloquinoline quinone (PQQ)-dependent ethanol dehydrogenase and *rd* for reductive dehalogenase, and the highest gene abundance was observed for *exaA* (Figure 3B). The stimulation of these gene families indicated that not only reductive dehologenation genes/pathways, but also hydroxylation and methoxylation genes/pathways may be involved in the BDE-209 degradation.

### Shifts of the Functional Genes Involved in Energy Process

**Figure 4:**
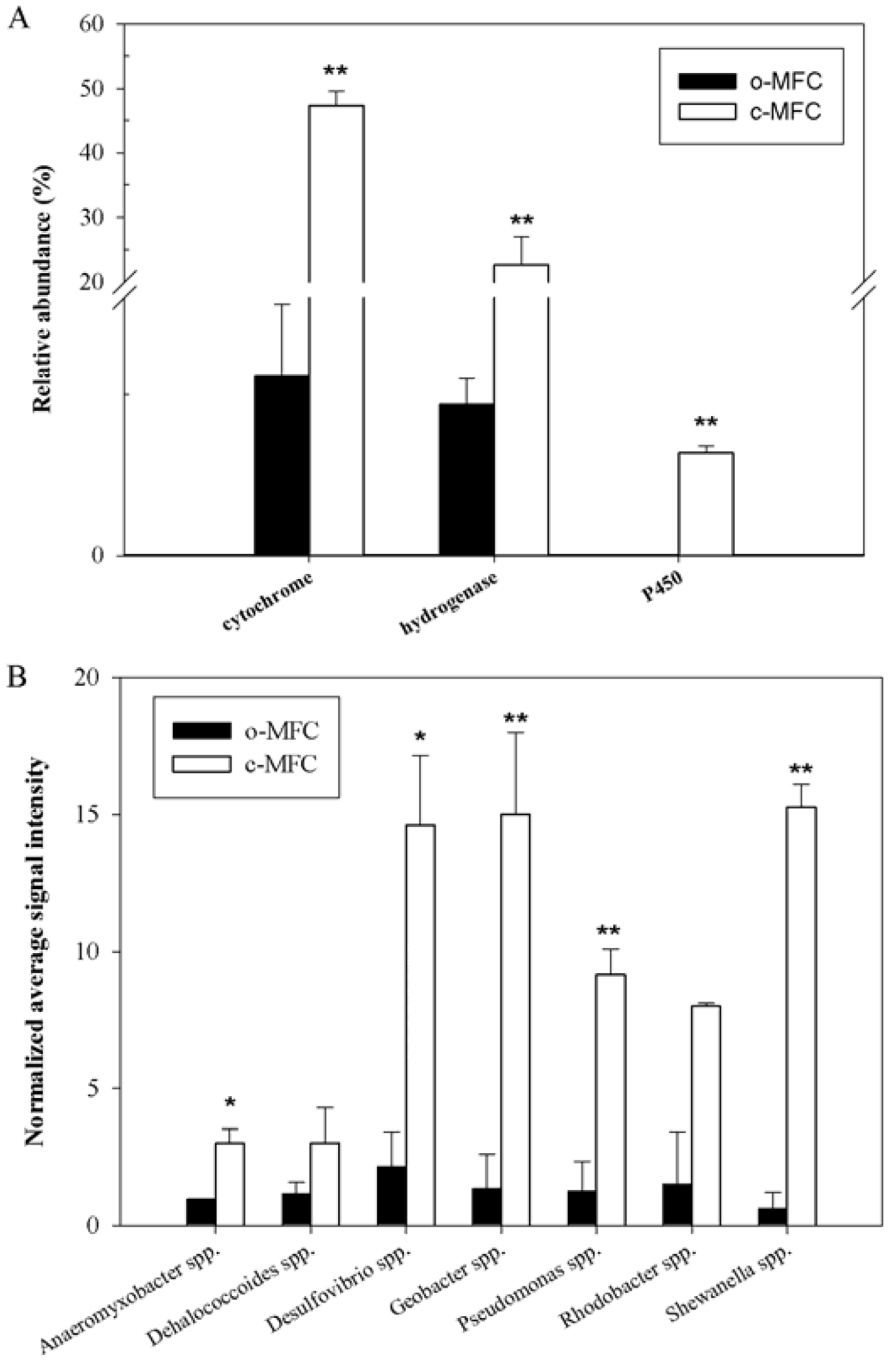
The normalized signal intensity of the detected key gens families (A) and the organisms (B) involved in energy transformation process under both c-MFCs and o-MFCs. The signal intensities were the sum of detected individual gene sequences for each functional gene, averaged among three samples. All data are presented as mean±SE. **p<0.05, *p<0.10.

Among the genes involved in energy process detected, the relative abundances of cytochrome genes and hydrogenase genes showed significant (*p*<0.05) increase in c-MFCs (Figure 4A). Among 106 genes involved in energy process, 91 genes were unique to the c-MFC samples and only three hydrogenase genes from *Desulfovibrio vulgaris* RCH1 (241879224, 241880985) and *Anaeromyxobacter* sp. K (197124673) were unique to o-MFCs. Seven P450 genes detected were all unique to c-MFC samples, and one with the highest signal intensity was from *Burkholderia* sp. 383 (78062539). Based on response ratio, cytochrome *c* class I genes from *Geobacter* sp. M21 (191162597) and *Pseudomonas putida* GB-1 (167034533) were significantly (*p*<0.05) increased in c-MFCs.

All detected energy process genes belonged to 13 genera, among which seven genera were shared by both c-MFC and o-MFC samples, and six were unique to c-MFCs. Although no significant difference was observed from the known dehalogenating bacteria, *Dehalococcoides* spp., the three significant (*p*<0.05) enriched anodic respiring genera in c-MFCs (Figure 4B), *Geobacter* spp., *Pseudomonas* spp. and *Shewanella* spp., are all capable of aromatic degradation and dechlorination. These results suggest that the introduction of electrode may stimulate the versatile functional microbes for current generation and PBDE degradation.

### The Composition of PBDE Congener as a Predominant Factor Shaping Microbial Community Functional Structure

**Figure 5:**
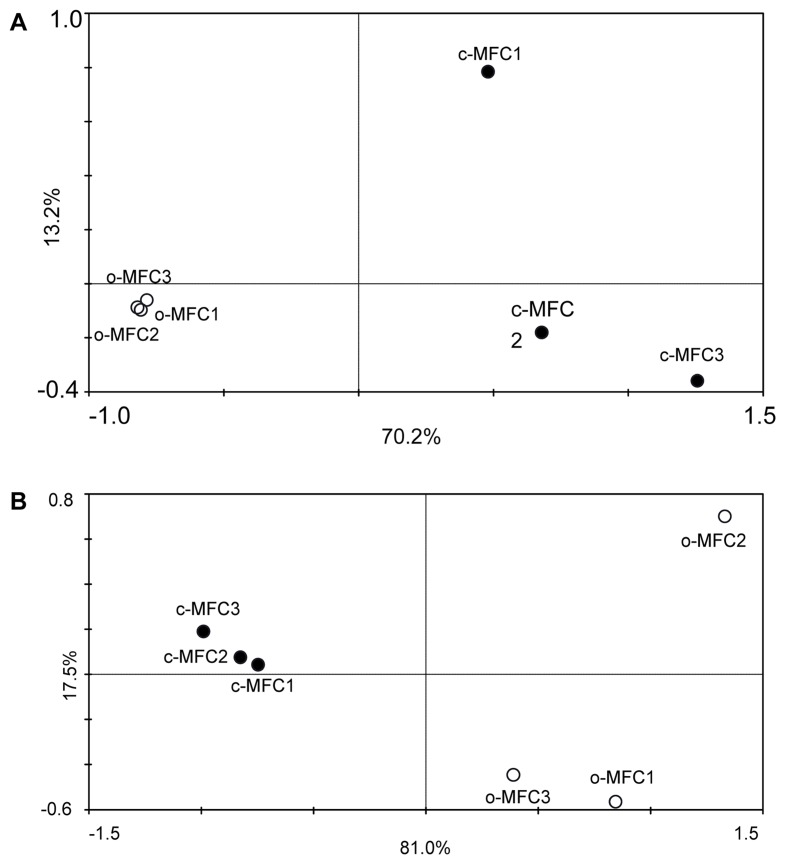
Principal-component analyses (PCA) of entire functional gene communities (A) and PBDE congeners (B) detected on day 70. Open circles represent samples collected from o-MFC systems and solid circles represent samples collected from c-MFC systems.

To determine the correlations between the microbial community functional structure and the BDE-209 degradation, PCA was performed based on all detected functional genes, different functional gene categories and PBDE congeners, respectively. All of the samples from the c-MFCs and o-MFCs were well separated along PC1 when the functional genes detected were used as the species (Figure 5A). However, the opposite patterns were observed when the PBDE congeners were used as the variables (Figure 5B). These results suggested that the microbial functional gene compositions were significantly influenced by the microbial electricity generation and their abundances were increased with the degradation of PBDE congeners.

In order to determine the most significant factors shaping the microbial community structure, CCA combined with forward selection procedure and variance inflation factors was conducted to assess the relationships between microbial community structure, electricity generation, PBDE congener compositions and hydrochemical parameters in the systems. Consistent with PCA results, no matter the entire functional gene community or the gene categories for chlorinated solvent remediation or aromatic degradation were used as the species, the samples from c-MFCs and o-MFCs were well separated by the first axis which could explained more than 60% of the total variances. On the basis of variance in inflation factors, three variables were selected: electric voltage, the concentration of BDE-183 and BDE-206. The specified CCA model was significant (*p = *0.018). Furthermore, these three factors could significantly (*p = *0.032) explain the functional genes involved in chlorinated solvent remediation and aromatic degradation.

**Figure 6:**
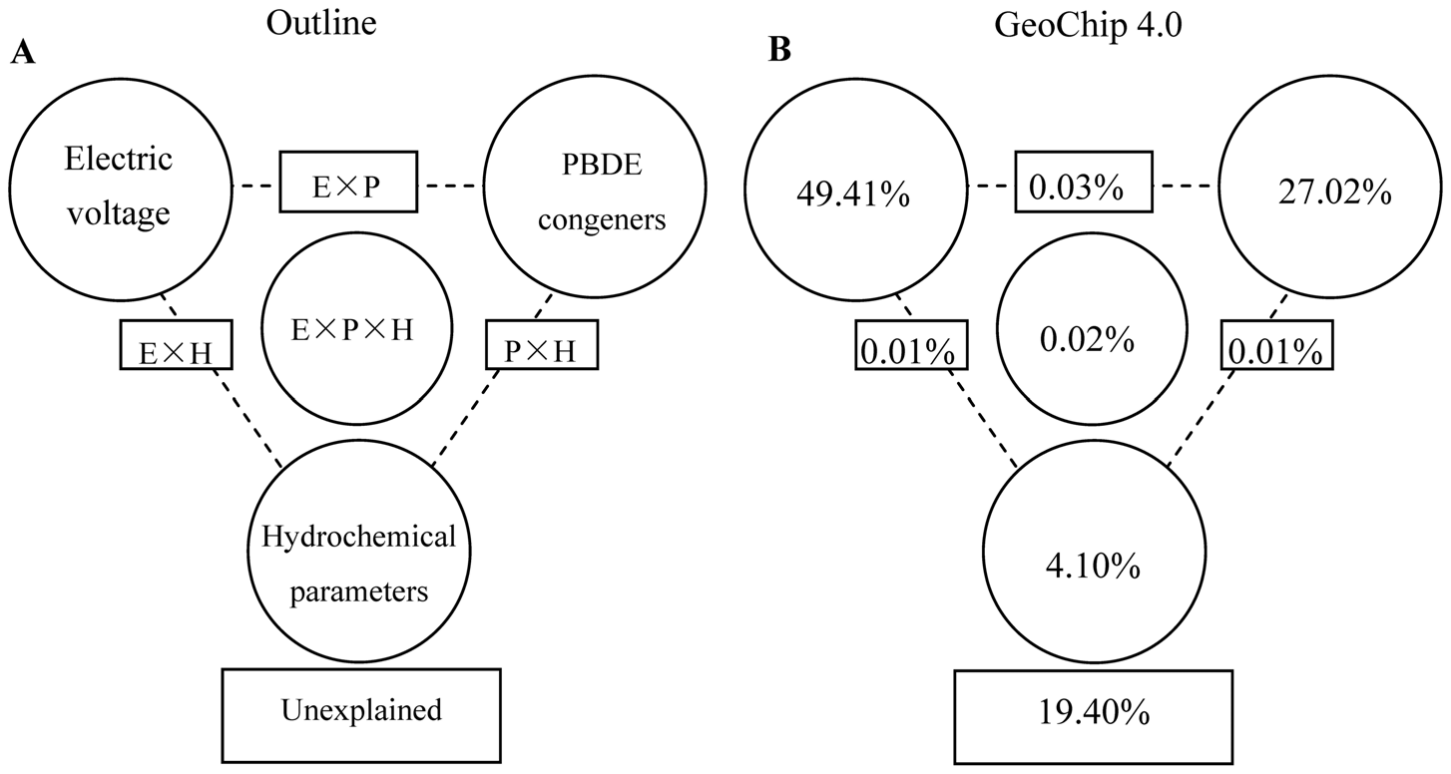
Variation partitioning based on CCA for all functional gene signal intensities. (A) General outline. (B) All functional genes. A CCA-based VIF was performed to identify the variables important to the microbial community structure. PBDE congener variables included BDE183, BDE-206 and BDE-208. Hydrochemical parameters included pH and the concentration of bromine ion.

To separate the effects of electricity generation, PBDE congener composition and the hydrochemical parameters on microbial community structure after 70 days performance, a CCA-based variation partitioning analysis was conducted. The electric voltage, PBDE congener composition and hydrochemical parameters showed a significant (*p = *0.041) correlation with the microbial functional gene structure. Electric voltage explained substantially more variation (49.41%, *p = *0.001) than PBDE congener composition (27.02%, *p = *0.028), whereas hydrochemical parameters independently explained 4.10% (*p = *0.428) of the observed variation (Figure 6). About 19.40% of the functional community variation based on GeoChip data remained unexplained by the above selected variables. These results suggest that after 70 days performance the microbial electricity generation changed the microbial community structure and accelerated the PBDEs transformation, and the PBDE congeners conversely acted as the predominant factor for shaping the functional microbial community.

## Discussion

The contamination of sediments and soils with persistent hydrophobic organic pollutants in large areas requires the development of effective bioremediation strategies. As the highly persistent hydrophobic organic pollutants, BDE-209 has been frequently detected in sediment and soil samples. It has been evidenced that BDE-209 could be slowly debrominated under anaerobic conditions by microorganisms. The amendments of trichloroethene as co-substrate [7], 4-bromobenzoic acid, 2,6-dibromobiphenyl, tetrabromobisphenol A or hexabromocyclododecane as primer compound [5] could significantly accelerate PBDE debromination. However, these strategies are unsuitable for bioremediation due to the toxicity of those added compounds. Our previous studies found that the amount of carbon source in sediment was enough to support PBDE debromination and the addition of exogenous electron donors could change the microbial community structure, as well as the PBDE congener compositions, but no significant decrease in total PBDE congeners was obtained [9], [18].

More and more studies have demonstrated that the lack of suitable electron acceptors was the most limiting factor for biodegradation of organic pollutants in anaerobic sedimentary environments [27], [28]. The addition of electron acceptors has been applied as the promising strategies to stimulate the abundances and activities of the indigenous microorganisms for *in situ* anaerobic bioremediation of contaminated aquifers [29]. To date, chemical components, such as Fe(III), nitrate and sulfate, have been widely used to accelerate anaerobic biodegradation rate. However, all of these components are soluble, very easy to diffuse away from the point of application, and inappropriate for bioremediation of persistent organic pollutants, such as PBDEs, which require a long-term treatment. Electrodes, which could be permanently placed at a specific point of application, offer a low-maintenance source electron acceptor for promoting anaerobic biodegradation of organic contaminants [13], [14]. In this study, our results clearly showed that the functional microbial gene abundances and PBDE transformations could be significantly enhanced with simultaneous electricity generation, suggesting that the bioremediation of the persistent hydrophobic organic pollutants in anaerobic sedimentary conditions could be accelerated when microbial electricity was generated. It is interesting that the composition and amount of PBDE congeners generated from BDE-209 degradation were much less than our previous studies [9]. Since the same analyzing methods were used in those studies, it is reasonable to presume that compounds other than PBDE congeners were generated in this study which means new microbial BDE-209 degradation pathways might be involved. Some other enzymatic reactions such as hydroxylation or methoxylation of PBDEs have been observed in animals, birds, fishes and algae [30]. It is possible that similar reactions can be catalyzed by microorganisms, as suggested by the functional gene results in this study. Further researches are needed to confirm this hypothesis.

To date, very few PBDE-degrading microbial strains were isolated. Given the structural similarity between PCBs and PBDEs, some known dechlorinators have been used to degrade the PBDEs, such as *Rhodococcus jostii* RHA1 [17], *Sphingomonas* sp. PH-07 [16] and *Dehalococcoides* sp. [7]. Although the involvements of some known dechlorinators in PBDE transformation were evidenced, very little information about the enzymes necessary to achieve PBDE biodegradation has been reported. Until now, only the typical dioxygenases required for PCB degradation have been identified to be involved in transformation of PBDEs by using reverse transcription quantitative PCR and gene recombinant analyses in a pure culture system [16], [17]. However, more and more evidence indicates that it is possible that an entirely different enzyme or multiple enzymes are responsible for PBDE transformation [17].

GeoChip, as a comprehensive functional gene array, has been proven to be an ideal tool for analyzing microbial communities and linking their structure with environmental factors as well as ecosystem functioning [20], [21], [31], [32], [33]. In this study, GeoChip 4.0 was used to detect the functional microbial community composition and structure for PBDE transformation in c-MFC and o-MFC systems. Our results clearly showed that most important functional genes, including those involved in chlorinated solvent remediation, aromatic degradation and energy transformation process, were significantly stimulated and BDE-209 degradation was dramatically enhanced with simultaneous electricity generation. The significant positive correlations were generally observed between a range of functional gene categories and the removal rates of PBDEs, indicating that PBDE degradation process is complex and a variety of functional genes could be involved in PBDE transformation. Interestingly, for functional genes involved in chlorinated solvent remediation, not only those genes encoding reductive dehalogenase, but also those encoding methyltransferase and dehydrogenase were significantly increased in c-MFCs. These results suggest that reductive dehologenation, hydroxylation and methoxylation may be all involved in the degradation of BDE-209. The total signal intensities of most important microbes, which are capable of not only dechlorination but also anodic respiration, significantly increased in c-MFCs, suggesting that the introduction of electrode provided the condition for microbial electricity generation and enhance PBDE degradation. It will be necessary to combined some open format metagenome technologies to identify the new members and pathways involved in PBDE degradation during microbial electricity generation, since GeoChip can only detect the genes contained in the array.

In summary, our results indicate that PBDE degradation could be stimulated with the significant increases of a variety of microbial functional genes after 70 days performance with electrode as the electron acceptor to produce microbial electricity. Our results also imply that PBDE biodegradation is a complex process in which reductive debromination, hydroxylation, and methoxylation, as well as aromatic degradation, may be all involved simultaneously. These findings strongly support the core hypothesis that the degradation of persistent organic pollutants in the anaerobic environments could be stimulated by the introduction of electrodes for microbial electricity generation. However, to elucidate the detailed PBDE biodegradation pathways under microbial electricity generation condition, it is essential to launch an integrated and comprehensive monitoring program to track the dynamics and adaptive responses of microbial communities to PBDE contamination together with other physical and chemical analysis in terms of microbial electricity generation, PBDE transformation and composition of their products.

## Supporting Information

File S1 **Supporting information files. Data S1** method: Response ratio (RR). **Data S2** Results: Shifts of the functional genes involved in carbon, nitrogen, sulfur and phosphorus cycles. **Table S1** Summary of the functional genes detected. **Table S2** The gene involved in aromatic degradation. **Figure S1** Voltage output (A) and polarization curve (B) of c-MFCs. **Figure S2** The normalized signal intensity of the detected key gene families involved in sulfur cycling under both c-MFC and o-MFC systems. The signal intensities were the sum of detected individual gene sequences for each functional gene, averaged among three samples. All data are presented as mean±SE. **p<0.05, *p<0.10. **Figure S3** The normalized signal intensity of the detected key gene families involved in phosphorus cycling under both c-MFC and o-MFC systems. The signal intensities were the sum of detected individual gene sequences for each functional gene, averaged among three samples. All data are presented as mean±SE. **p<0.05, *p<0.10. **Figure S4** Principal-component analyses (PCA) of entire functional gene communities (A) and PBDE congeners (B) detected. Open circles represent samples collected from o-MFC systems and solid circles represent samples collected from c-MFC systems. **Figure S5** Ordination plot produced from redundancy analysis (RDA) of entire functional gene communities on day 70 detected using GeoChip 4.0. Open circles represent samples collected from o-MFC systems and solid circles represent samples collected from c-MFC systems. Three significant PBDE congeners, BDE183, BDE-206 and BDE-208, were selected by forward selection based VIF with 999 Monte Carlo permutations.
(DOCX)
