# Supplementary material for: Microbial Electricity Generation Enhances Decabromodiphenyl Ether (BDE-209) Degradation
Source: PLoS One. 2013 Aug 5;8(8):e70686. doi: 10.1371/journal.pone.0070686 (PMC3734261; doi:10.1371/journal.pone.0070686)
Supplement: File S1 — Supporting information files. Data S1 method: Response ratio (RR). Data S2 Results: Shifts of the functional genes involved in carbon, nitrogen, sulfur and phosphorus cycles. Table S1 Summary of the functional genes detected. Table S2 The gene involved in aromatic degradation. Figure S1 Voltage output (A) and polarization curve (B) of c-MFCs. Figure S2 The normalized signal intensity of the detected key gene families involved in sulfur cycling under both c-MFC and o-MFC systems. The signal intensities were the sum of detected individual gene sequences for each functional gene, averaged among three samples. All data are presented as mean±SE. **p<0.05, *p<0.10. Figure S3 The normalized signal intensity of the detected key gene families involved in phosphorus cycling under both c-MFC and o-MFC systems. The signal intensities were the sum of detected individual gene sequences for each functional gene, averaged among three samples. All data are presented as mean±SE. **p<0.05, *p<0.10. Figure S4 Principal-component analyses (PCA) of entire functional gene communities (A) and PBDE congeners (B) detected. Open circles represent samples collected from o-MFC systems and solid circles represent samples collected from c-MFC systems. Figure S5 Ordination plot produced from redundancy analysis (RDA) of entire functional gene communities on day 70 detected using GeoChip 4.0. Open circles represent samples collected from o-MFC systems and solid circles represent samples collected from c-MFC systems. Three significant PBDE congeners, BDE183, BDE-206 and BDE-208, were selected by forward selection based VIF with 999 Monte Carlo permutations. (DOCX) [file pone.0070686.s001.docx]

**Supporting information**

**Data S1** Method:

Response ratio (RR)

**Data S2** Results:

Shifts of the functional genes involved in carbon, nitrogen, sulfur and phosphorus cycles

**Table S1** Summary of the functional genes detected.

**Table S2** The gene involved in aromatic degradation.

**Figure S1** Voltage output (A) and polarization curve (B) of c-MFCs.

**Figure S2** The normalized signal intensity of the detected key gene families involved in sulfur cycling under both c-MFC and o-MFC systems. The signal intensities were the sum of detected individual gene sequences for each functional gene, averaged among three samples. All data are presented as mean±SE. **p<0.05, *p<0.10.

**Figure S3** The normalized signal intensity of the detected key gene families involved in phosphorus cycling under both c-MFC and o-MFC systems. The signal intensities were the sum of detected individual gene sequences for each functional gene, averaged among three samples. All data are presented as mean±SE. **p<0.05, *p<0.10.

**Figure S4** Principal-component analyses (PCA) of entire functional gene communities (A) and PBDE congeners (B) detected. Open circles represent samples collected from o-MFC systems and solid circles represent samples collected from c-MFC systems.

**Figure S5** Ordination plot produced from redundancy analysis (RDA) of entire functional gene communities on day 70 detected using GeoChip 4.0. Open circles represent samples collected from o-MFC systems and solid circles represent samples collected from c-MFC systems. Three significant PBDE congeners, BDE183, BDE-206 and BDE-208, were selected by forward selection based VIF with 999 Monte Carlo permutations.

**Data S1** Method:

**Response ratio (RR)**

The response ratio (RR) is a statistical test as described in the reference (Luo et al., Ecology, 2006, 87, 53-63). Briefly, the mean and standard deviation (SD) of each treatment were extracted. The RR of each variable was calculated by dividing the mean of the treatment group to that of the control group. The variances for all comparisons were estimated by the means, the standard deviations, and the sample sizes in treatment and control groups, which were used to calculate the standard errors for each RR. Based on the standard error, the 90% or 95% confident interval for each response variable was obtained and the statistical difference between the c-MFCs and o-MFCs was estimated. For the response ratio analysis, the total abundance of each gene category or family was simply the sum of the normalized intensity for the gene category or family.

**Data S2** Results:

**Shifts of the functional genes involved in carbon, nitrogen, sulfur and phosphorus cycles**

*Carbon degradation*. Among the carbon cycling genes detected, more than 86.2% involved in carbon degradation, such as starch, hemicellulose, cellulose, chitin and lignin. The carbon degradation gene number detected in c-MFCs was around 10.9 times of that in o-MFCs, and only 24 genes were shared by both MFC systems. Almost all of the carbon degradation genes showed significantly (*p* < 0.05) higher abundances in c-MFCs than in o-MFCs (Figure S2). For 24 shared genes, 19 genes showed significant (*p* < 0.05) changes and only one *xylA* gene for hemicellulose degradation from *Dinoroseobacter shibae* DFL 12 (159044542) decreased in c-MFCs. In addition, for 20 genes involved in methane cycling, 18 genes were unique to the c-MFC samples with 7 *mcrA* genes for the *α* subunit of methyl coenzyme M reductase, 11 *pmoA* genes for methane monooxygenase, 2 *mmoX* for particulate methane monooxygenase, and only one *mcrA* gene from *Methanocorpusculum labreanum* Z (124363917) was detected in the o-MFCs.

*Nitrogen cycling.* Totally, 745 nitrogen cycling genes were detected and belonged to 15 gene families involved in assimilatory N reduction, dissimilatory N reduction to ammonium, N_2_ fixation, denitrification processes, and ammonification (Figure S3). Among those 15 gene families detected, 12 of them had significantly (*p* < 0.05) higher abundances in c-MFCs than in o-MFCs, while *norB* for nitric oxide reductase and *nosZ* for nitrous oxide reductase did not show significant differences between cMFC and oMFC samples. Totally, 416 denitrifying genes were detected in this study and most of them were from uncultured bacteria. Among 27 shared denitrifying genes by cMFC and oMFC samples, nine genes showed significant changes based on the response ratios with six *narG* genes (78093536, 78093518, 209401612, 228015257, 199594017 and 32307929), one *nirS* (77378473) and one *nirK* (83316878) increased and only one *narG* gene (62003537) decreased in c-MFCs. Furthermore, the percentages of the functional genes involved in denitrification decreased along the process (NO_3_^-^→NO_2_^-^→NO^-^→N_2_O^-^→N_2_). Interestingly, *hzo* genes encoding hydrazine oxidoreductase involved in the anammox process were only detected in c-MFCs and no nitrification genes (e.g., *amoA*, *hao*) were detected in both systems, suggesting that the anammox process could be promoted to drive the ammonium oxidization under microbial electricity generation conditions.

*Sulfur.* Higher number and abundance of the genes involved in sulfur cycling were detected in c-MFCs (Figure S4). For the detected 113 *dsrA/B* genes encoding dissimilatory sulfite reductase, 98 were unique to c-MFCs and four were unique to o-MFCs. Among 11 shared *dsrA/B* genes, two (20502025 and 46520023) were significantly (*p* < 0.05) increased in c-MFCs based on the RR analysis. Most of the detected genes were derived from uncultured microorganisms, while sulfate-reducing populations related to *Caldivirga*, *Chlorobaculum*, *Chlorobium*, *Clostridium*, *Desulfacinum*, *Desulfoarculus*, *Desulfofaba*, *Desulfohalobium*, *Desulfospira*, *Desulfotomaculum*, *Halochromatium*, *Magnetospirillum*, *Mitsuokella*, *Syntrophobacter*, and *Thiobacillus* were only detected in c-MFC and *Pyrobaculum* species were only detected in o-MFCs. Among 56 *sox* genes encoding the sulfur-reducing enzyme system, 50 of them were from c-MFC systems while only two genes were shared by both systems with one from *Methylobacterium* sp. 4-46 (149120191) significantly increased in c-MFCs. These results suggested that the functional genes involved in sulfur cycling could be enhanced when coupled with microbial electricity generation.

*Phosphorus*. Totally, 86 genes involved in phosphorus cycling were detected, and more than 82.1% of them were unique to c-MFC systems. The total abundance of *ppk* genes encoding polyphosphate kinase in c-MFCs was significant (*p* < 0.05) higher than that in o-MFCs (Figure S5). For six shared P cycling genes, only one gene (170777715) from *Leptothrix cholodnii* SP-6 encoding exopolyphosphatase (*ppx*) for inorganic polyphosphate degradation was detected with significantly (*p* < 0.01) increased abundance in c-MFCs. The three genes encoding phytase for phytate degradation were from *Aspergillus niger*, *Buttiauxella* sp. GC21, and *cf. Ceriporia* sp. CBS 100231, and they were unique to c-MFC. These results suggested that phosphorus utilization could be stimulated in c-MFC systems.

| **Gene category** | **Gene number detected** | | | | **Gene abundance detected** | | | |
| --- | --- | --- | --- | --- | --- | --- | --- | --- |
|  | **c-MFCs** | **o-MFCs** | **c-MFC/ o-MFCs** *^a^* | **Response-ratio***^b^* | **c-MFCs** | **o-MFCs** | **c-MFC/ o-MFCs** *^a^* | **Response-ratio***^b^* |
| Total genes | 5646.5±9.2 | 638.3±56.9 | 9.7 | 2.3 | 6213±981 | 638±57 | 8.5 | 2.1 |
| Carbon cycling | 700.0±14.1 | 72.7±3.2 | 10.6 | 2.4 | 696.7±28.9 | 85.3±2.1 | 9.4 | 2.2 |
| Nitrogen cycling | 457.5±13.4 | 52.3±3.2 | 9.6 | 2.3 | 450.0±16.3 | 65.1±10.4 | 8.1 | 2.1 |
| Energy process | 67.0±1.4 | 8.7±2.1 | 8.6 | 2.2 | 67.5±0.04 | 8.7±2.6 | 9.1 | 2.2 |
| Metal resistance | 697.5±13.4 | 90.0±15.7 | 8.5 | 2.1 | 755.5±35.1 | 117.4±26.5 | 7.4 | 2.0 |
| Organic remediation | 1657.5±21.5 | 173.0±20.8 | 10.5 | 2.4 | 1700.8±48.4 | 210.9±31.8 | 9.4 | 2.2 |
| Phosphorus | 64.5±0.7 | 11.3±2.5 | 6.7 | 1.9 | 70.3±7.1 | 16.6±8.9 | 4.9 | 1.6 |
| Sulphur | 163.5±6.4 | 19.0±.0 | 9.8 | 2.3 | 143.2±3.5 | 31.8±14.2 | 5.4 | 1.7 |
| Other genes | 572.0±1.4 | 66.3±2.3 | 9.4 | 2.2 | 588.9±13.9 | 80.3±12.4 | 8.4 | 2.1 |

**Table S1 Summary of the functional genes detected**

*^a^* The average ratio.

*^b^* Response ratios of the genes detected under current generation are all significant at *p*<0.01.

**Table S2 The gene involved in aromatic degradation**

| **Gene category** | **Unique genes number** | | | **Shared genes number** | | |
| --- | --- | --- | --- | --- | --- | --- |
|  | **Total** | **c-MFC** | **o-MFC** | **Total** | **Significant increase (*p*<0.05)***^a^* | **Significant decrease (*p*<0.05)***^a^* |
| Aromatic carboxylic acid | 930 | 888 | 42 | 81 | 20 | 1 |
| BTEX and related aromatics | 105 | 98 | 7 | 5 | 0 | 0 |
| Chlorinated aromatics | 105 | 103 | 2 | 12 | 4 | 0 |
| Heterocyclic aromatics | 23 | 23 | 0 | 23 | 0 | 0 |
| Nitroaromatics | 145 | 137 | 8 | 13 | 1 | 0 |
| Other aromatics | 398 | 382 | 16 | 36 | 13 | 0 |
| Polycyclic aromatics | 126 | 124 | 2 | 11 | 5 | 1 |

*a***:** The genes number with significant changes in response ratio.

**Figure S1** Voltage output (A) and polarization curve (B) of c-MFCs.


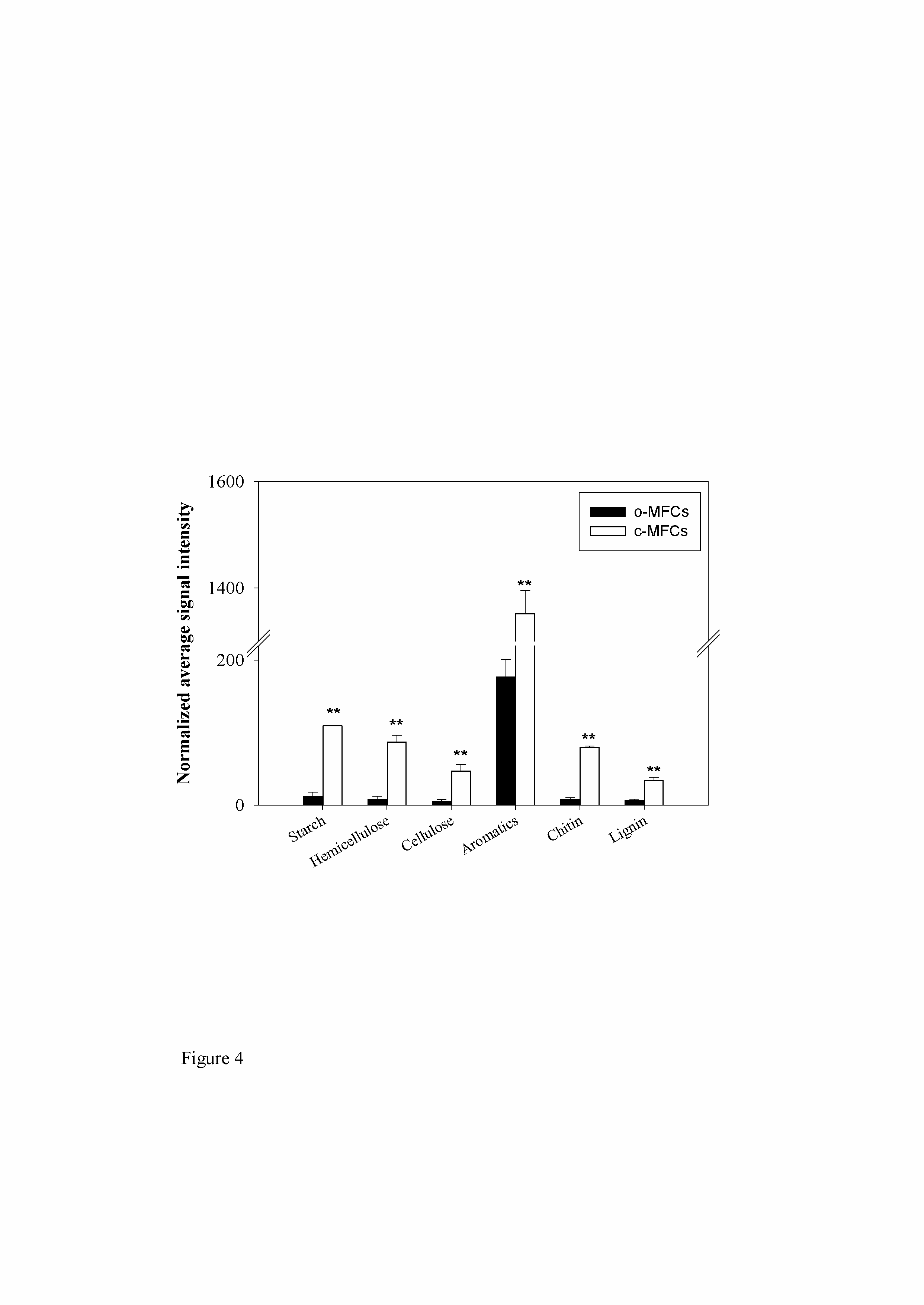

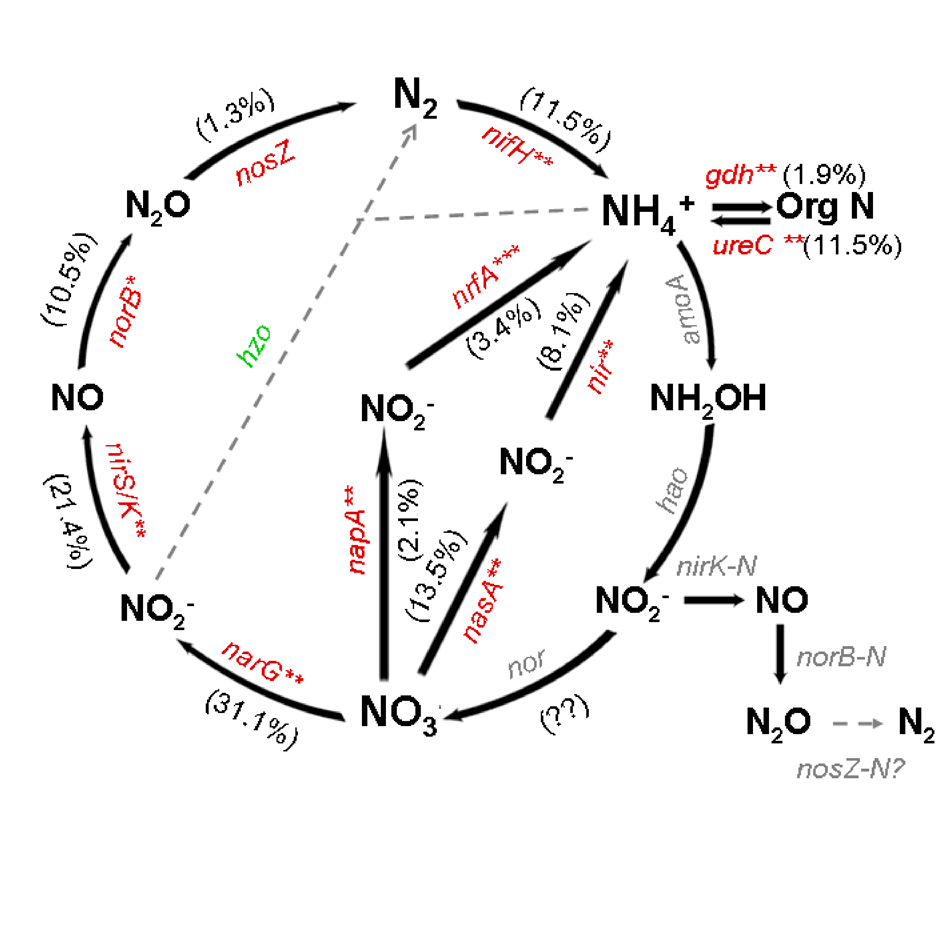


**Figure S2** The normalized signal intensity of the detected key gens families involved in carbon cycling under both c-MFCs and o-MFCs. The signal intensities were the sum of detected individual gene sequences for each functional gene, averaged among three samples. All data are presented as mean±SE. ***p*<0.05.

Figure S4 The relative changes of the detected genes involved in nitrogen cycling at c-MFC. The signal intensity for each gene detected was normalized by all detected gene sequences using the mean. The percentage of a functional gene in a bracket was the sum of the signal intensity of all detected sequences of this gene divided by the grand sum of the signal intensity of the detected nitrogen cycling genes, and weighted by the fold change of the signal intensity of this gene at c-MFC to that at o-MFC. For each functional gene, colours mean that this gene had a higher (red) signal intensity at c-MFC than at o-MFC with significance at *p*<0.05 (**).Gray-coloured genes were not targeted by this GeoChip, or not detected in those samples. The green-colured gene (*hzo*) was unique to c-MFC. It remains unknown if *nosZ* homologs exist in nitrifiers.


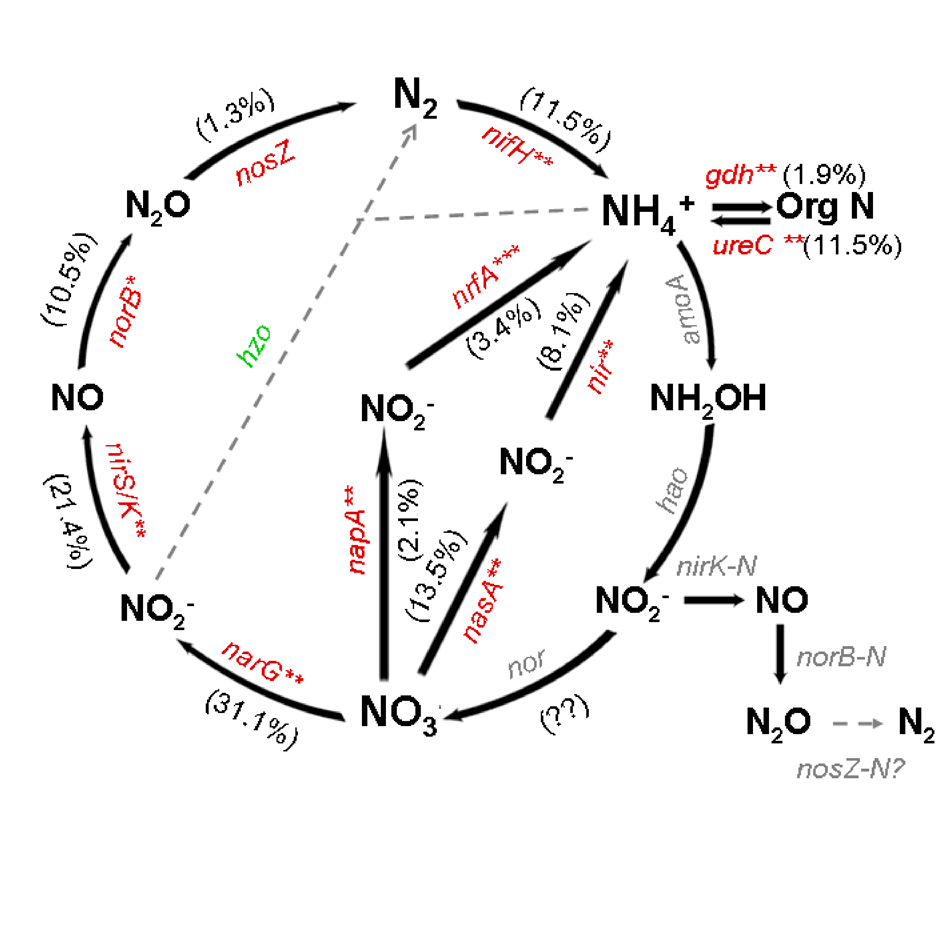


**Figure S3** The relative changes of the detected genes involved in nitrogen cycling at c-MFC. The signal intensity for each gene detected was normalized by all detected gene sequences using the mean. The percentage of a functional gene in a bracket was the sum of the signal intensity of all detected sequences of this gene divided by the grand sum of the signal intensity of the detected nitrogen cycling genes, and weighted by the fold change of the signal intensity of this gene at c-MFC to that at o-MFC. For each functional gene, colours mean that this gene had a higher (red) signal intensity at c-MFC than at o-MFC with significance at *p*<0.05 (**).Gray-coloured genes were not targeted by this GeoChip, or not detected in those samples. The green-colured gene (*hzo*) was unique to c-MFC. It remains unknown if *nosZ* homologs exist in nitrifiers.


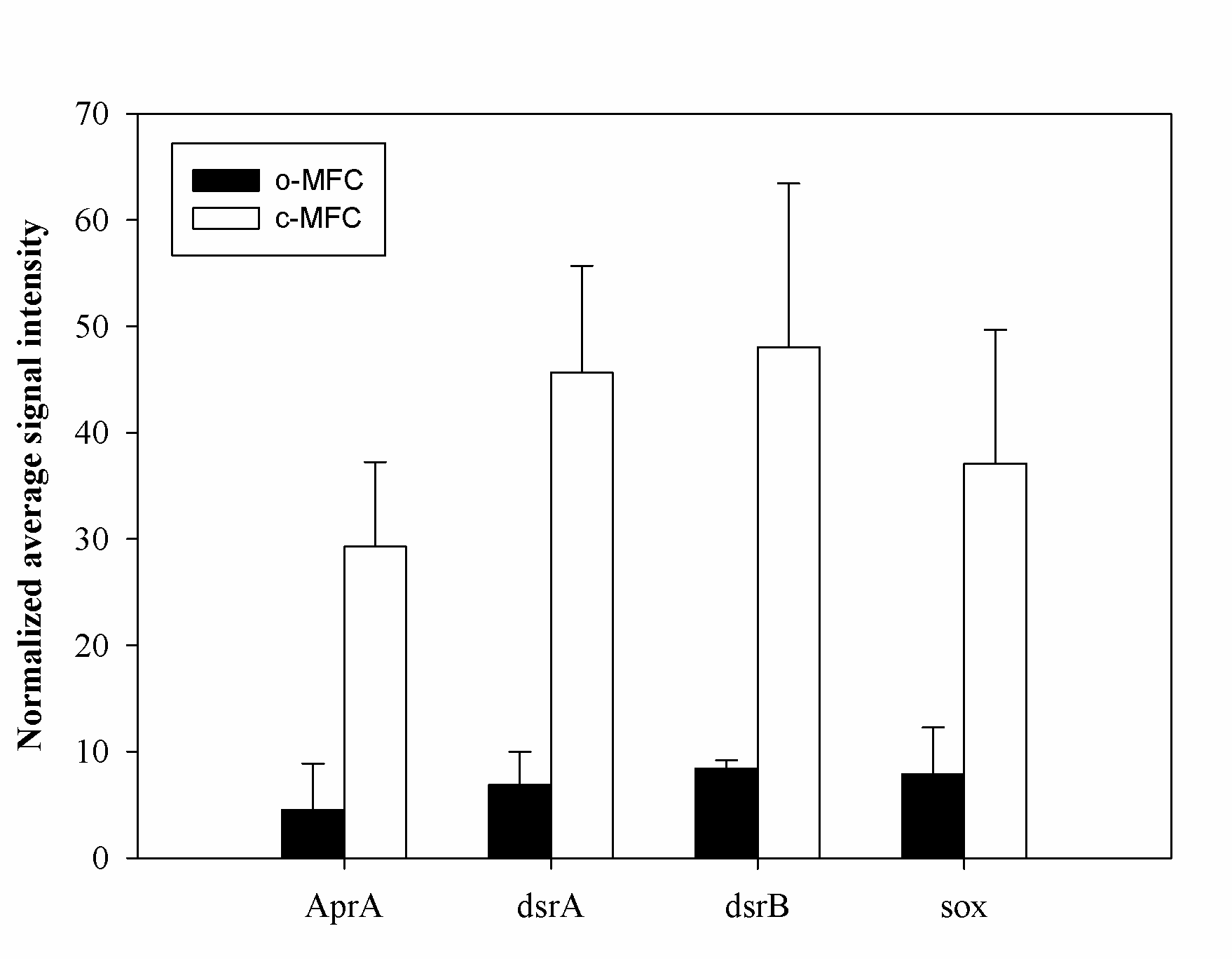


**

**

*

*

**A**

**A**

**Figure S4** The normalized signal intensity of the detected key gene families involved in sulfur cycling under both c-MFC and o-MFC systems. The signal intensities were the sum of detected individual gene sequences for each functional gene, averaged among three samples. All data are presented as mean±SE. ***p*<0.05, **p*<0.10.


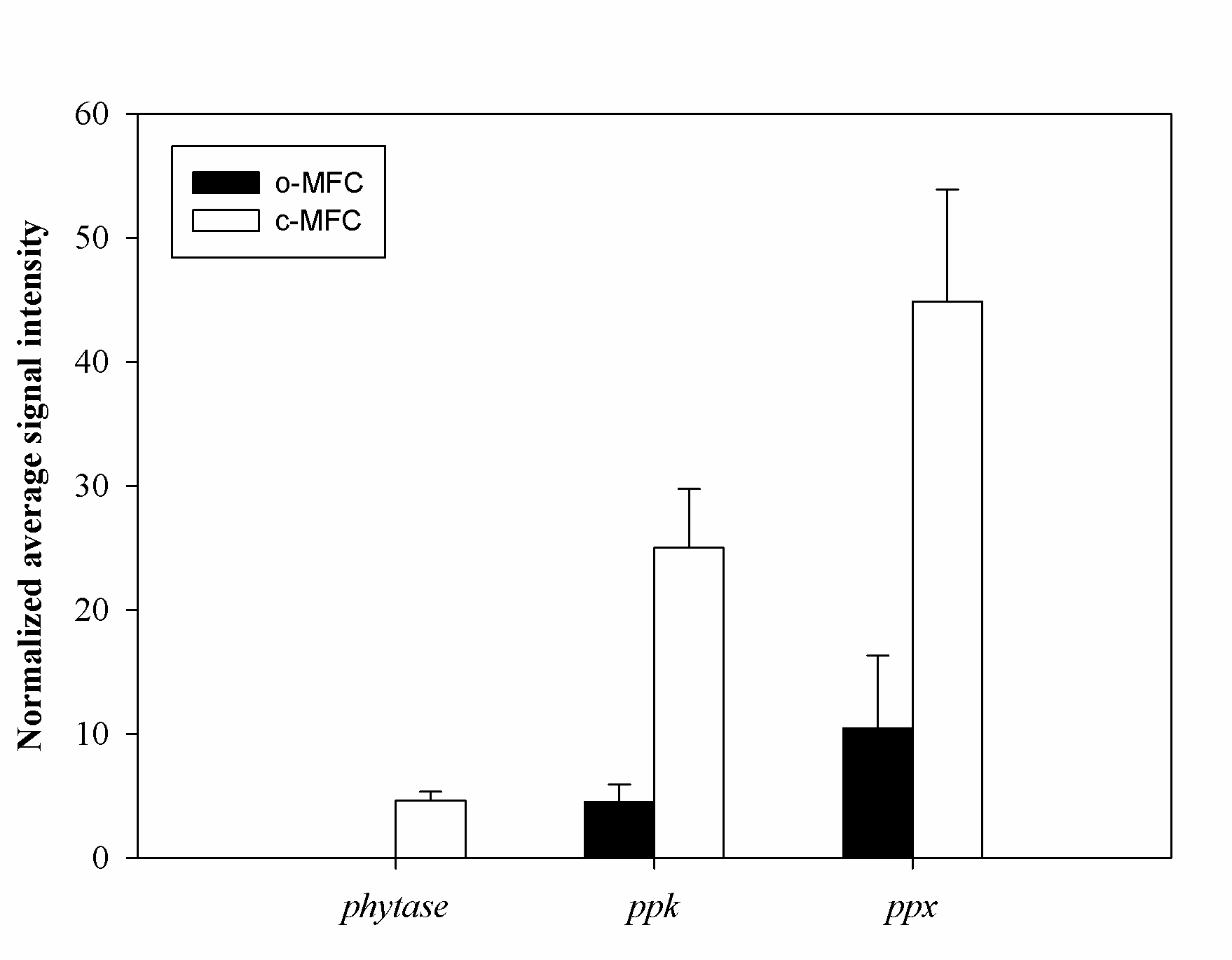


**

*

**Figure S5** The normalized signal intensity of the detected key gene families involved in phosphorus cycling under both c-MFC and o-MFC systems. The signal intensities were the sum of detected individual gene sequences for each functional gene, averaged among three samples. All data are presented as mean±SE. ***p*<0.05, **p*<0.10.
